# The pro-angiogenic role of hypoxia inducible factor stabilizer FG-4592 and its application in an in vivo tissue engineering chamber model

**DOI:** 10.1038/s41598-019-41924-5

**Published:** 2019-04-15

**Authors:** Muran Zhou, Jinfei Hou, Yuan Li, Shan Mou, Zhenxing Wang, Raymund E. Horch, Jiaming Sun, Quan Yuan

**Affiliations:** 1 Department of Plastic Surgery, Union Hospital, Tongji Medical College, Huazhong University of Science and Technology, 1277 JieFang Avenue, Wuhan, 430022 China; 2 Department of Plastic and Hand Surgery, University Hospital of Erlangen, Friedrich Alexander University, Erlangen Nuernberg, (FAU), Erlangen, Germany

## Abstract

Tissue engineering is a promising technology used as an alternative to organ/tissue transplantation which is often limited by donor shortage. The construction of large-sized engineered tissue requires a fast and sufficient vascularization process. Previous studies have shown that hypoxia-inducible factor (HIF) -1α may promote the vascularization process implying that stabilized HIF-1α can be applied in the engineering of large-sized tissue. However, the toxicity and off-target effect of previously reported HIF-1α stabilizers limit their clinical application. FG-4592, a small molecule specific HIF stabilizer, was previously investigated as an anti-anemia drug in a phase-III clinical trial. Here we found that FG-4592 promoted tube formation in an *in vitro* model of angiogenesis by stabilizing HIF-1α and activating vascular endothelial growth factor (VEGF). When FG-4592 immobilized fibrin gel scaffold was implanted into a subcutaneous tissue engineering chamber, the vascularization process was significantly enhanced through the similar mechanisms which was verified *in vitro*. We conclude that FG-4592 may serve as a pro-angiogenic molecule for the construction of large-sized engineered tissue where intensive angiogenesis is required.

## Introduction

Although tissue transplantation remains to be the preferred therapy for tissue defects, the shortage of donors is a major obstacle that limits its application. Since its first report in 1987, tissue engineering is now a widely used technology which presents an alternative approach to overcome the problem of donor shortage^1^. However, for large three-dimensional tissue engineered structures, lack of vascularization limits the survival of the seeded cells at the center of the structures^2^. Thus, it is important to develop strategies that facilitate the angiogenic process in tissue engineered structures. Incorporation of angiogenic growth factors such as vascular endothelial growth factor (VEGF), basic fibroblast growth factor (BFGF) and platelet-derived growth factor (PDGF) into the tissue engineered construct is a popular strategy to promote vascularization^3,4^. However, the application of a single growth factor only leads to malformed and leaky vessels^5^. Hypoxia Inducible Factors (HIF), the master regulator of oxygen homeostasis^6^, play a key role in angiogenesis^7^. More than 100 angiogenic growth factors, including VEGF, BFGF, and PDGF were found to be downstream targets of HIF^8^. Therefore, upregulating HIF might be a superior strategy to promote the angiogenic process.

**Figure 1:**
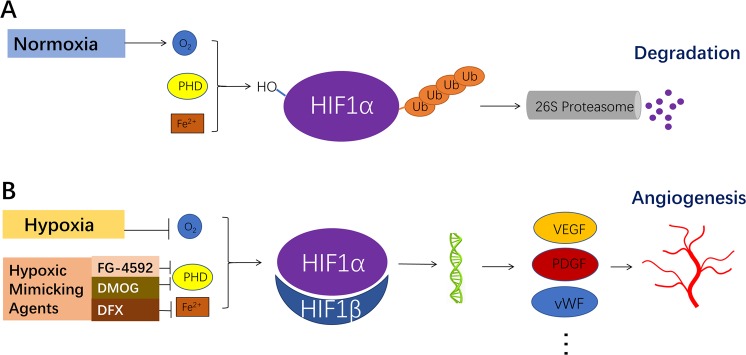
The regulation of HIF-1α. (**A**) In normoxic condition, Prolyl hydroxylase (PHD), in the presence of Fe^2+^ and oxygen molecule, hydroxylates HIF-1α and triggers its further ubiquitination and proteolysis. (**B**) In hypoxic condition or with the help of hypoxic mimicking agents such as FG-4592, the hydroxylation and degradation of HIF-1α are blocked. Thus HIF-1α can dimerize with HIF-1β, enter the nucleus to transcriptionally regulate the expression of its target genes such as VEGF, PDGF and vWF, ultimately regulating a wide range of pathophysiologic processes including angiogenesis.

The oxygen-dependent, Prolyl hydroxylase domain-containing proteins (PHDs)-mediated regulatory mechanisms of HIF have been extensively described by several research groups^9^. (Fig. 1 and Supplementary Video 1). HIF-1, which is heterodimerized from α and β subunits^10^, is the most widely expressed HIF isoform which plays a critical role in hypoxic response^8,11^. Though the constant expression of HIF-1α, it would be degraded within 5 minutes under normoxic condition^12^. In the presence of oxygen, divalent iron ions (Fe^2+^) and ascorbate, PHDs hydroxylate the proline (Pro) or asparagine (Asn) residues in HIF-1α and triggering ubiquitination and proteolysis of HIF-1α^13,14^. In hypoxic condition, HIF-1α escapes hydroxylation by PHDs and dimerizes with HIF-1β subunit. The heterodimerized HIF-1 further binds to its target genes, thereby regulating their transcription.

The level of HIF-1α is regulated by intracellular oxygen level in several pathophysiological conditions through an adaptive response. Drugs that increase the expression of HIF are called hypoxic mimicking agents. Previous studies reported hypoxic mimicking agents including divalent iron ions antagonists such as CoCl_2_ and deferoxamine (DFX) or competitive inhibitors of PHDs such as Dimethyloxallyl Glycine(DMOG)^15–17^. FG-4592, a novel small-molecule HIF-PHD inhibitor, was developed using structure-based drug design (SBDD) and high throughput screening (HTS) technologies. It is currently under phase III clinical trials for adult patients with renal anemia^18^. Compared to traditional hypoxia mimicking agents, FG-4592 has higher target specificity and is safer for clinical application^19^. Considering its hypoxia-inducible factor stabilizing effect, we hypothesized that FG-4592 may promote the vascularization process and serve as a pro-angiogenic molecule in tissue engineering and other fields where angiogenesis is required.

## Materials and Methods

### Cell culture

Human umbilical vein endothelial cells (HUVECs) were used for the *in vitro* bio-functional study on FG-4592. The primary HUVECs were purchased from Sciencell and cultured with Endothelial Cell medium (ECM) supplemented with 10% fetal bovine serum (FBS), 1% EC growth supplement (ECGS), 1% antibiotics (100IU penicillin and 0.1mg streptomycin per ml). Further experiments were carried out with cells at passage four to six. For the *in vitro* biosafety study, RAW264.7 cell line was used to test inflammatory response after FG-4592 treatment. RAW264.7 were purchased from China Center for Type Culture Collection (CCTCC) and cultured with high glucose DMEM medium supplemented with 10% FBS, 1% antibiotics for cell expansion and replaced with low serum (1% FBS) medium in preparation for further analysis.

### Wound healing assay

HUVECs were seeded on 12-well plates and cultured to 100% confluence. This was followed by introduction of scratches on the cell monolayer with a 100 ul pipette tip to ensure a constant width. Cells were then incubated with ECM containing 0 uM, 5 uM, 20 uM, 50 uM FG-4592 (Selleckchem, Houston, USA), respectively. The cell migration process was observed at 0, 6, 12, 18 hours using a bright-field microscope (OLYMPUS CKX41).

### Tube formation assay

Tube formation assay was performed by adding 50ul Matrigel (BD Biosciences, Bedford, MA, USA) into each well of a 96-well plate and then polymerizing for 30 min at 37 °C. HUVECs suspended in ECM containing 0 uM, 5 uM, 20 uM or 50 uM FG-4592, were plated at a density of 1 × 10^4^/well. After incubating for 8 hours, images were captured with a bright-field microscope (OLYMPUS CKX41). To analyze tube formation quantitatively, the length of branching and the amount of enclosed polygonal structures in the digital images were analyzed using Image-Pro Plus 6.0 software (Media Cybernetics, Rockville, MD).

### Quantitative real-time PCR

Quantitative real-time PCR was performed to test the expression of HIF-1α and VEGF1 in HUVECs as well as IL-1β and TNF-α in RAW264.7 cell line. Briefly, when HUVECs reached 60% confluence on the six-well plate, they were treated with 3ml ECM containing 0 uM or 20 uM FG-4592 for 48 hours. Meanwhile, when RAW264.7 reached a 70% confluence on six-well plates, they were treated with 3ml high-glucose DMEM containing 0 uM or 20 uM FG-4592 for 16 hours. Total RNA was extracted using a miRNeasy Micro Kit according to the manufacturer’s instruction. Briefly, 2 ug of total RNA was reverse transcribed into cDNA using a Revert Aid First Strand cDNA Synthesis Kit (Thermo). Real-time quantitative PCR was carried out using FastStart Universal SYBR Green Master (Roche) on 7300 Real-Time PCR system (Applied Biosystems) according to the manufacturer’s protocol. The reactions were run in a 96-well optical plate at 95 °C for 15 min, followed by 40 cycles of 95 °C for 15 sec, 55  °C for 30 sec and 70 °C for 30 sec. The melting program consisted of 95 °C for 15 sec, 60 °C for 30 sec and 95 °C for 15 sec. All the reactions were performed in triplicate. The PCR products cycle threshold (Ct) data were obtained using fixed threshold settings. A comparative Ct method was used to analyze the mRNA expression in the samples. The primers used are displayed in Table 1.

**Table 1:** Nucleotide sequences of the primers used for Q-rtPCR analysis.

Gene	Primer sequence
H-ACTIN-S	CACCCAGCACAATGAAGATCAAGAT
H-ACTIN-A	CCAGTTTTTAAATCCTGAGTCAAGC
H-HIF1α-S	TGATTGCATCTCCATCTCCTACC
H-HIF1α-A	GACTCAAAGCGACAGATAACACG
H-VEGF-S	GGAGGGCAGAATCATCACGA
H-VEGF-A	GCTCATCTCTCCTATGTGCTGG
M-Tnf-F	GGCCAACGGCATGGATCT
M-Tnf-R	GGCAGCCTTGTCCCTTGAA
M-Il1b-F	ATGGGCTGGACTGTTTCTAATG
M-Il1b-R	CTTGTGACCCTGAGCGACC
M-GAPDH-F	GACAAAATGGTGAAGGTCGGT
M-GAPDH-R	GAGGTCAATGAAGGGGTCG

### Western Blot

Cultured HUVECs to 60% confluence on six-well plate were treated with 3ml ECM containing 0 uM or 20 uM FG-4592 for 48 hours and then lysed in cold RIPA buffer (Servicebio, Wuhan, PRC). The protein concentration was determined using the BCA Protein Assay Kit (Servicebio). Proteins were separated by 8% SDS-PAGE, transferred to a PVDF membrane (Millipore), and then blocked in 5% nonfat dried milk in TBST for 1 hour. The PVDF membrane was then incubated with rabbit monoclonal antibody against HIF-1α (1:1000, Servicebio), and VEGF (1:1000, Servicebio), or rabbit antibody against β-Actin (1:1000, Servicebio) as an internal reference overnight at 4 °C, and then washed with TBST for 5 min, 3 times. Subsequently, the PVDF membrane was incubated with mouse anti-rabbit horseradish-peroxidase-conjugated secondary antibody (1:3000, Servicebio) for 30 min at room temperature. After washing with TBST for 15 min, the immune complexes on the PVDF membrane were detected using an Immobilon TM Western Chemiluminescent Kit (Servicebio). Image Pro plus software 6.0 was used to analyze the relative protein expression, represented as the density ratio to β-Actin.

### Isolation chamber and fibrin matrix

**Figure 2:**
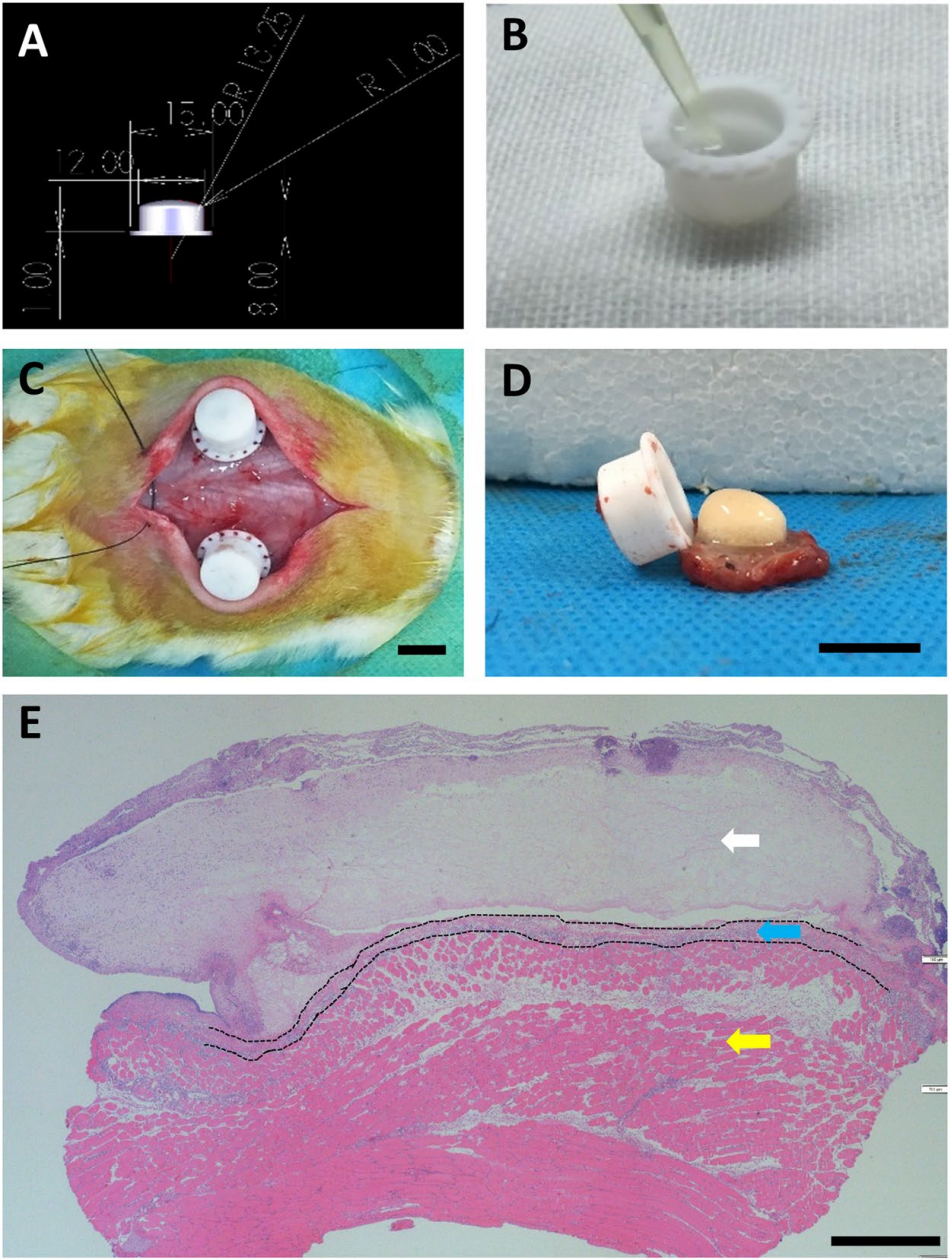
The construction of tissue engineering chamber model. Teflon isolation chamber with a 12 mm inner diameter and 8 mm height (**A**) filled with FG-4592 loading fibrin gel (**B**) was embedded on the dorsal muscle of SD rats (**C**). The chamber together with fibrin clots and surrounding tissues was explanted (**D**) and subjected to various histochemistry staining as shown. HE-stained explanted tissue exhibited a fibrin clot (white arrows), dorsal muscle (yellow arrows) and adjacent fibrovascular tissue (blue arrows) growing from the muscle towards the fibrin clot (**E**) Scale bar: **C**,**D **= 10 mm; E = 500 um.

The cylindrical chamber was 10 mm in inner diameter and 8mm in height, and was made of heat resistant medical grade Polytetrafluoroethene (PTFE) (Fig. 2A). A clinically approved human fibrin sealant kit (RAAS, ShangHai, PRC) with a final concentration of 10 mg/ml fibrinogen, 16 IU/ml Thrombin and 1500 KIU/ml Aprotinin (Solarbio, Beijing, PRC) was used to prepare a fibrin matrix(Fig. 2B). The matrix was loaded with FG-4592 in 3 different concentrations: negative control (NC) = 0 mM; low dosage (LD) = 24 mM; high dosage (HD) = 48 mM (Table 2) which were polymerized before implantation (Supplementary Video 2).

### Animals

Twenty-seven syngenic male Sprague-Dawley rats (250–300 g) purchased from the Experimental Animal Center of Tongji Medical College, Huazhong University of Science and Technology (HUST) were used in this study. These pathogen-free rats were kept in standardized animal house with free access to standard chow and water and maintained on a 12 hours light/dark cycle at a stable temperature. All animal experiments were approved by the Animal Care and Use Committee of HUST, and were performed in compliance with the National Institutes of Health Guide for the Care and Use of Laboratory Animals. Group design for the animal study was summarized in Table 2.

**Table 2:** Experimental design of the *in vivo* study.

Time	3 days	7 days	14 days
FG-4592 0 mM (NC)	6 constructs	6 constructs	6 constructs
FG-4592 24 mM (LD)	6 constructs	6 constructs	6 constructs
FG-4592 48 mM (HD)	6 constructs	6 constructs	6 constructs

### Surgical procedures

All operations were performed under anesthesia with 2% Isoflurane inhalation by the same surgeon. A midline skin incision was made on the back of the rats to produce a large subcutaneous pocket. Two chambers containing 600ul fibrin matrix were symmetrically bilaterally implanted in the subcutaneous pocket with the opening facing the dorsal muscle. The chambers were fixed using Prolene 3–0 sutures (Ethicon, Norderstedt, Germany) (Fig. 2C). After hemostasis and skin closure, all rats were given 0.1ml Benzylpenicillin-Benzathine (Tardomycel Comp®, Bayer, Leverkusen, Germany) intramuscularly.

### Histological and immunohistochemical analysis

According to the group design, fibrin matrix together with the surrounding tissue was explanted and fixed in 4% paraformaldehyde (Fig. 2D) at 3, 7 and 14 days postoperatively. Meanwhile, the kidneys were also explanted to study the global effect of FG-4592. Thereafter, the specimens were subjected to graded ethanol dehydration and then embedded in paraffin. Three-micron cross-sections of the central plane were stained with hematoxylin and eosin to evaluate the general morphology and the height of the fibrovascular tissue (Fig. 2E). Immuno-histochemical staining was performed to detect the expression of HIF-1α, VEGF, CD31, MAC2 and Ki67. Briefly, after antigen retrieval, the specimen were incubated with the primary antibody at 4 °C overnight. Primary antibodies used are as follow: goat anti-CD31 (santa, sc-1506, 1:50), rabbit anti-Ki67 (abcam, ab16667, 1:200), rabbit anti-HIF-1α (GeneTex, 1:200), rabbit anti VEGF (ptg, 19003-1-ap, 1:200), goat anti-mac2 (rd, af1154, 1:200). The sections were then washed with TBS and incubated with secondary antibody for 25 min at room temperature. Finally, color development was performed with diaminobenzidine (Dako Denmark A/S) as the substrate. The whole field view of HE-stained slices and five randomly selected 200X fields in each IHC stained slice were captured using a light microscope (Olympus IX71). The photos were analyzed with Image-Pro Plus 6.0 software.

### Statistical analysis

All data were presented as means ± standard deviation. Statistical analysis was performed using one-way analysis of Variance by GraphPad Prism 6.02 (GraphPad Software, Inc). *P* < 0.05 was chosen as the critical level of statistical significance.

## Results

### FG-4592 promotes migration and tube formation in HUVECs

**Figure 3:**
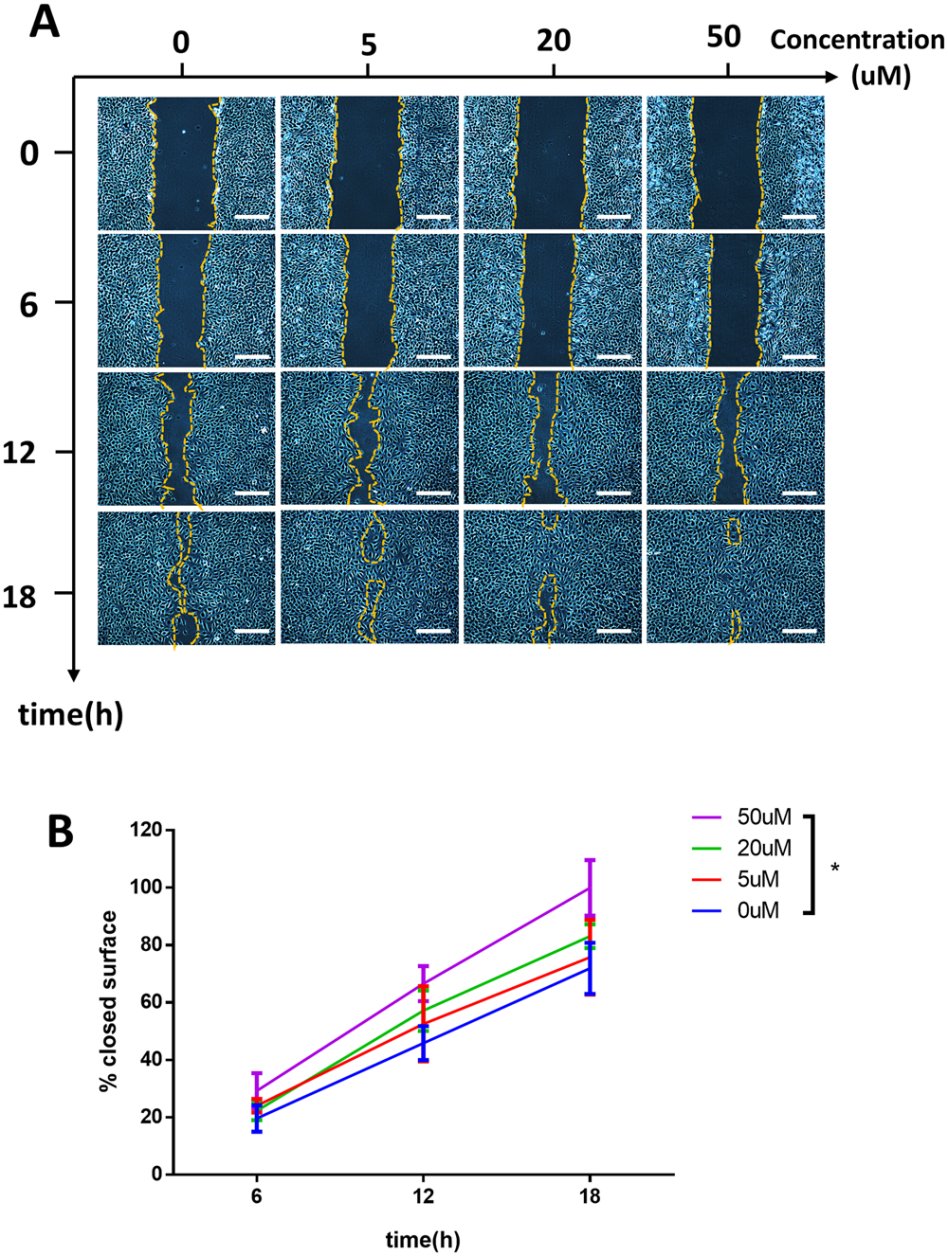
FG-4592 promotes migration of HUVECs in a dose-dependent manner. (**A**) Wound healing assay in HUVECS incubated with or without FG-4592 as evaluated by inverted optical microscopy. Scale bar = 250 um. (**B**) Quantitative analysis of relative cell migration. The closed surface percentage among the 4 groups at three different times is shown in the histogram. Significant differences between 50 uM FG-4592 treated group and negative control were observed at all three times. *p < 0.05.

Blood vessel formation is a physiological process which is strongly dependent on vascular endothelial cell migration^20^. Thus HUVECs wound healing assay was performed to explore the effect of FG-4592 on cell migration. We found that FG-4592 containing ECM promoted endothelial cell migration in a dose-dependent manner. Untreated cells reached only 42% of wound closure at 12 hours after the scratch was performed, whereas cells treated with 5, 20 or 50 uM FG-4592 containing ECM achieved 52%, 57% or 67% wound closure, respectively. This trend was observed throughout the experiment period (Fig. 3).

**Figure 4:**
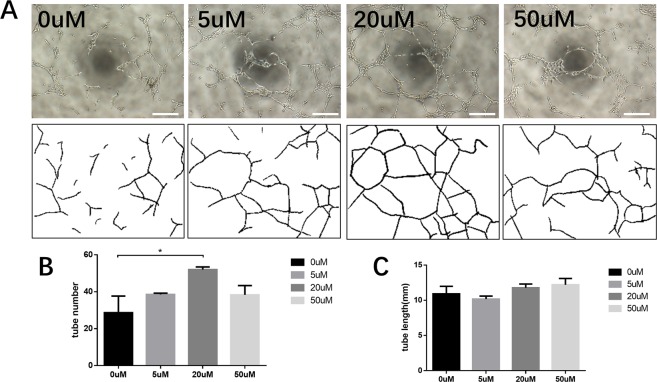
FG-4592 promoted angiogenesis *in vitro* (**A**). Representative tube formation images 8 hours after cell seeding. Scale bar = 250 um. (**B**) Quantitative analysis revealed that the administration of FG-4592 in the culture medium significantly increased the number of enclosed polygonal structures. (**C**) The branching length showed an elongation in drug-treated group. Each group was performed in three replicate wells and five randomly selected views from each well were captured to perform further analysis. *p < 0.05.

To further determine the effect of FG-4592 on capillary network formation *in vitro*, HUVECs tube formation assay was performed. We observed that the tube-like structure was more prominent in the drug-treated group as early as 4 hours after treatment and the peak tube formation occurred at 8 hours. The statistical analysis of images acquired at 8 hours posttreatment showed that the number of the tube-like structures was significantly higher in 20 uM FG-4592 containing ECM treated group compared to the negative control. A similar trend was observed in other drug-treated groups, although not significant (Fig. 4). This result suggests that at an appropriate concentration, FG-4592 may stimulate tube formation in HUVECs.

### The influence of FG-4592 on expression of PHD-HIF-VEGF axis and pro-inflammatory cytokines

**Figure 5:**
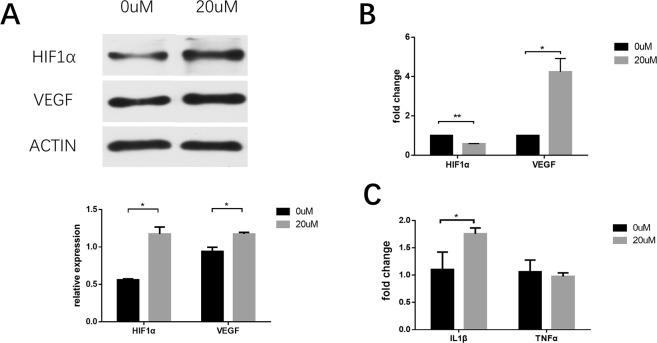
The influence of FG-4592 on expression of PHD-HIF-VEGF axis and pro-inflammatory cytokines. HUVECs or RAW264.7 were pretreated with 0 uM or 20 uM FG-4592 before samples were collected. (**A**) Western blot analysis of HIF-1α and VEGF protein expression in HUVECs after FG-4592 treatment. The relative expression of HIF-1α and VEGF was normalized to β-Actin. (**B**) Besides, Q-rtPCR analysis of HIF-1α and VEGF was performed. The relative mRNA expression was normalized to β-Actin and the expression of negative control group served as a reference, which was set as 1. (**C**) Similarly, Q-rtPCR analysis of TNFα and IL1β was performed to detect their expression in RAW264.7. *p < 0.05, **p < 0.01.

According to its mechanism of action, FG-4592 may influence the PHD-HIF-VEGF axis. Q-rtPCR and western blot were performed to explore this possibility at the transcription and translation level. We observed that pretreatment with FG-4592 containing ECM upregulated the expression of both HIF-1α and VEGF at the protein level in HUVECs. Interestingly, at the transcriptional level, VEGF mRNA expression was significantly higher in the drug-treated group, whereas HIF-1α mRNA expression was lower (Fig. 5A,B).

Considering the exogeneity and the HIF stabilizing effect of FG-4592, we evaluated its proinflammatory potential. After treatment with FG-4592 containing ECM, the transcriptional expression of proinflammatory cytokines TNF-α was unchanged whereas the expression of IL-1β was mildly elevated (Fig. 5C).

### Surgery and Animals

All the 27 animals tolerated the surgical procedure. Infection, hematoma, wound dehiscence or other major postoperative complications were not observed during the first week after surgery. No extrusion of the chamber occurred over the period of breeding. In the explant experiment of the chamber and subjacent muscular tissue, it was observed that there were newly formed capsules of fibrous tissue around the matrices containing chambers. After opening the capsules and chamber, the fibrin matrices were found to be adherent to the subjacent muscular tissue and the morphology of the fibrin matrices was substantially retained (Fig. 2D).

### The thickness of the fibrovascular tissue was increased by FG-4592 loading fibrin gel

**Figure 6:**
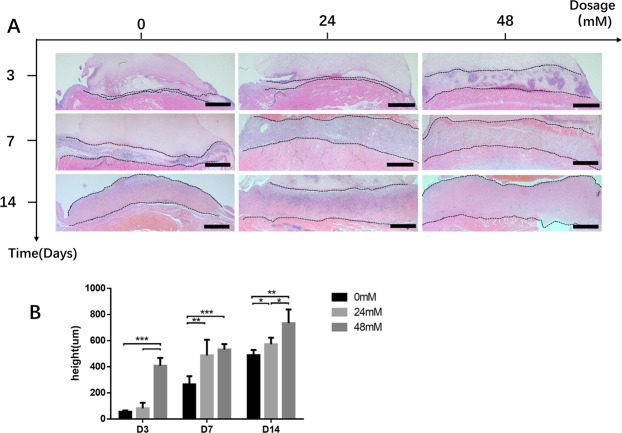
FG-4592 increased the thickness of fibrovascular tissue. (**A**) Thickness of the fibrovascular tissue at the HE-stained matrix-host interface of samples explanted 3 days, 7 days and 14 days after the initial operation. (**B**) Statistical analysis indicated that compared to the negative control (NC) group, FG-4592 at low dosage (LD) and high dosage (HD) increased the thickness of the fibrovascular tissue in a dose and time-dependent manner. Scale bar = 500 um. *p < 0.05, **p < 0.01, ***p < 0.001.

HE staining indicated that the fibrin gel scaffold had a homogeneous structure. During the 14 days of experiment, no vascular branching into the fibrin gel was observed. However, a stratum of fibrovascular tissue at the matrix-host interface containing microvasculatures and inflammatory cells was observed. Compared to the negative control, incorporation of FG-4592 in fibrin gel significantly increased the thickness of fibrovascular tissue in a dose-dependent manner at 14 days after implantation (HD = 734 ± 104 um; LD = 571 ± 51 um; NC = 488 ± 40 um). At other time points, this trend was also observed and the difference between HD group and NC groups was significant (Fig. 6).

### The maturation of neovasculature was promoted by FG-4592 loading fibrin gel

**Figure 7:**
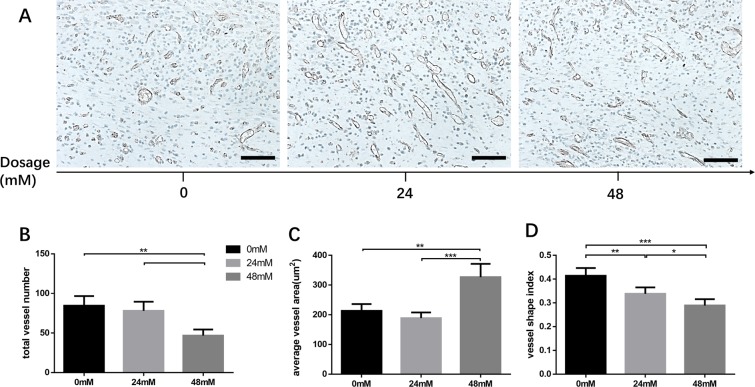
Representative images and morphometric analysis of CD31 immunohistochemical (IHC) staining sections. Morphometric analysis (B,C,D) performed based on CD31 staining images (**A**). Although the amount of neovasculature in the HD group was lower than in NC group (**B**), the neovasculature of HD group had a larger average vessel area (**C**). Furthermore, FG-4592 reduced vessel shape index in a dose-dependent manner, suggesting that neovasculature tended to grow upwards after drug treatment (**D**). *p < 0.05, **p < 0.01, ***p < 0.001. Scale bar = 100 um.

**Figure 8:**
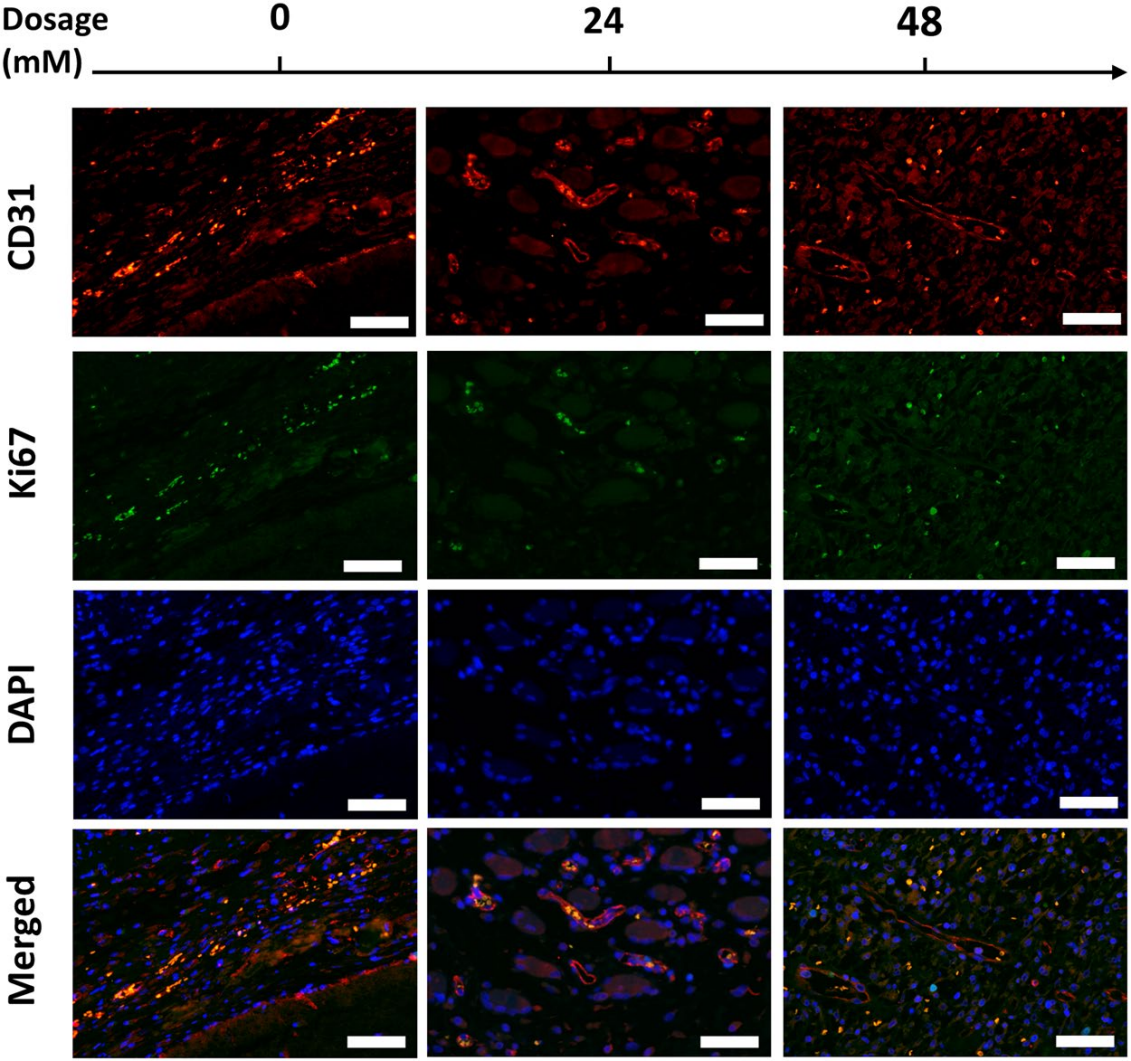
The attenuated endothelial cell proliferation after FG-4592 administration. The CD31 and Ki67 double staining immunofluorescence images showed that after FG-4592 administration, the number of CD31(+) Ki67(+) proliferating active endothelial cells was declined, which were observed as orange fluorescent cells around the vascular lumen in merged images. Scale bar: 50 um.

Total vessel number, average vessel area and vessel shape index (VSI) were used to evaluate the morphology of the vascular sections in this study. Using CD31 endothelial cell immunohistochemical staining, we found that the total vessel number in fibrovascular tissue in HD group was lower than that of NC group, whereas the average area of vessel tubule was larger. Besides, VSI decreased in the drug treatment group in a dose-dependent manner (Fig. 7). To investigate the cell proliferation status *in vivo*, Ki67-CD31 immunofluorescent staining was performed. The number of Ki67(+) CD31(+) positive cells in LD and HD group was lower than NC group, indicating that cell proliferation in fibrovascular tissue was reduced after drug administration (Fig. 8). Taken together, these data showed that the neovasculature after FG-4592 treatment would transform into more stable vascular with larger diameter, which indicated that FG-4592 promotes the maturation of neovasculature in this model.

### FG-4592 upregulated the protein expression of HIF-1α and VEGF *in vivo*

**Figure 9:**
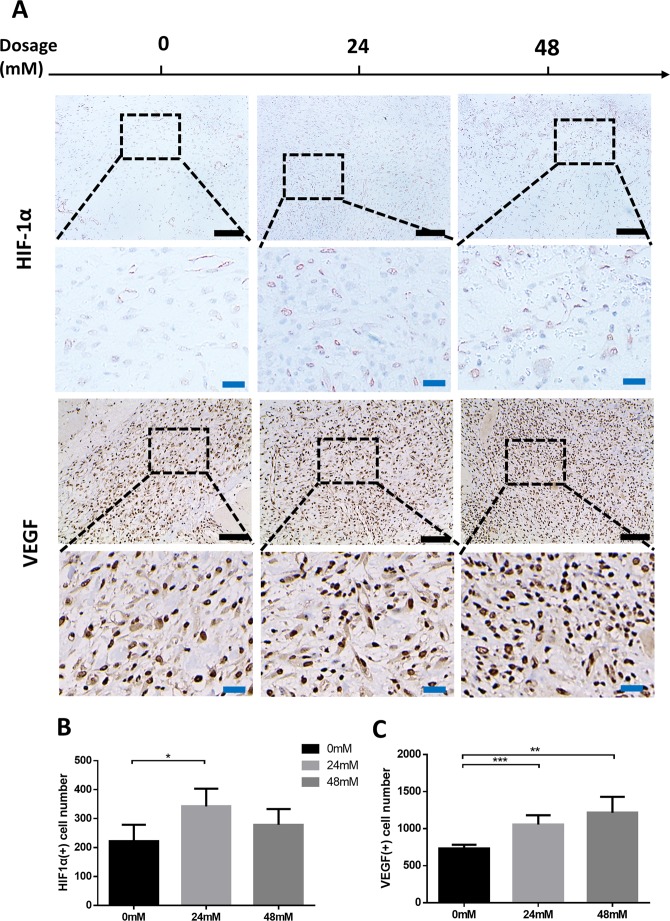
FG-4592 upregulated the expression of HIF-1α and VEGF *in vivo*. Fibrovascular tissue at the matrix-host interface was selected as the region of interest (ROI). Red stained HIF-1α (+) cells and diffused brown stained VEGF (+) cells were observed in all groups (**A**). In drug administered group (LD and HD), the numbers of HIF-1α (+) as well as VEGF (+) cell were higher than in negative control (**B**,**C**). Scale bar: black = 100 um, blue = 25 um.

To further explore the influence of FG-4592 on PHD-HIF1α-VEGF axis, we detected the expression of HIF-1α and VEGF at the protein level *in vivo* by immunohistochemical staining (Fig. 9A). The result showed that the number of VEGF positive cells in fibrovascular tissue was significantly higher in the drug-treated group compared to the negative control (Fig. 9C). HIF-1α displayed similar to VEGF but the difference between HD and NC group in HIF-1α expression was not statistically significant (Fig. 9B).

### The immunogenic potential and systemic effect of FG-4592 loading fibrin gel

**Figure 10:**
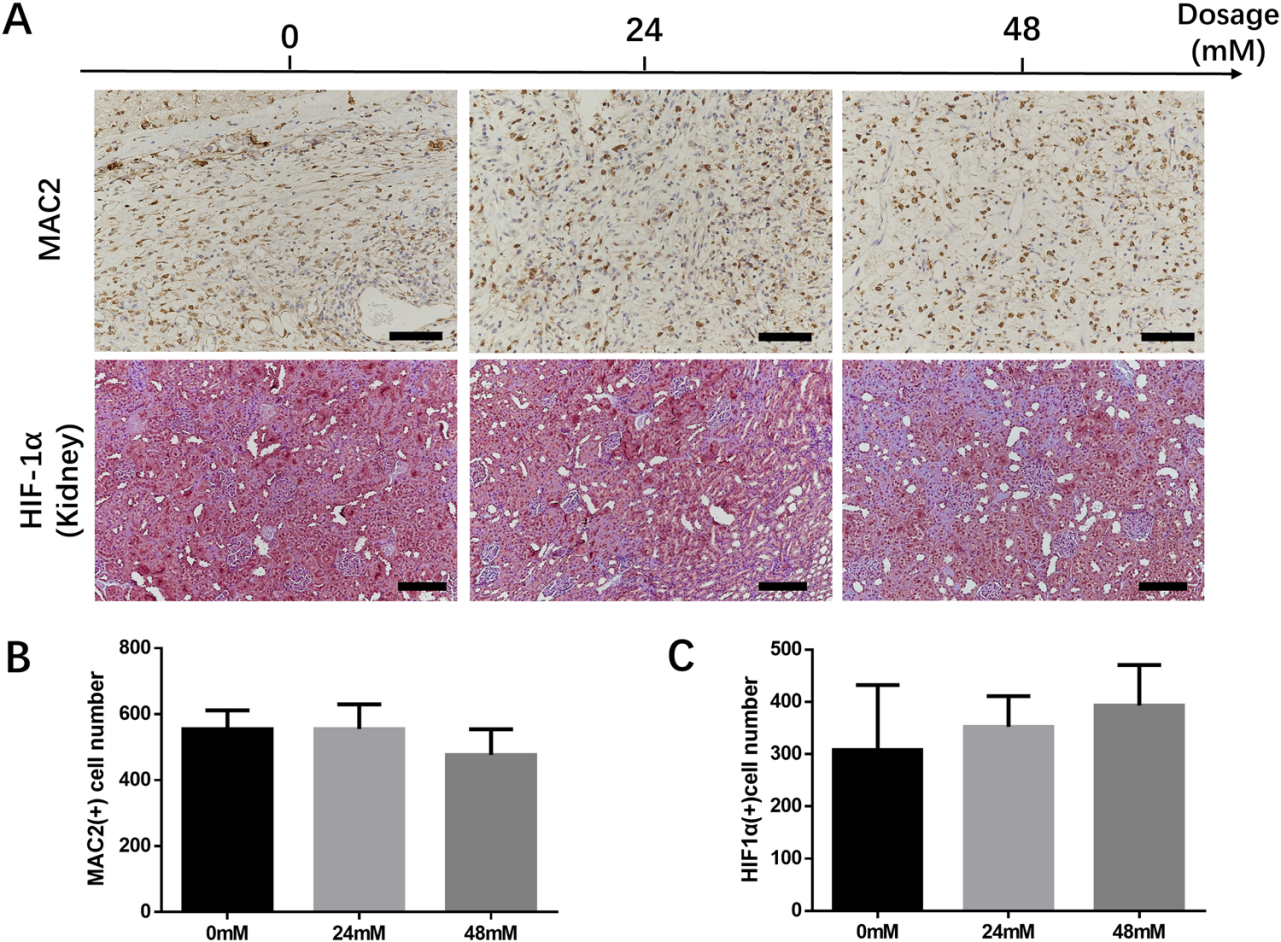
Locally administered FG-4592 does not promote inflammation or exert systemic effects. To examine inflammatory response, fibrovascular tissue at the matrix-host interface was selected as the region of interest (**A**). There were no significant differences in terms of MAC2 (+) cell numbers among the three groups (**B**). To examine the systemic effect, rat kidneys were used to perform HIF-1α staining (**A**) and no significant differences in terms of HIF-1α (+) cell numbers were found among the groups (**C**). Scale bar: black = 100 um, blue = 25 um.

To determine the immunogenic potential of FG-4592 using our *in vivo* tissue engineering chamber model, we performed MAC2 IHC staining to evaluate the inflammatory response based on macrophages (Fig. 10A). After loading different concentrations (24 mM and 48 mM) of FG-4592 in fibrin gel, the numbers of MAC2 (+) macrophages were not significantly changed compared with FG-4592 none treated group (Fig. 10B). This result suggested that FG-4592 loading fibrin gel does not trigger an inflammatory response *in vivo*. Although the purpose of our study is to verify the proangiogenic effect of FG-4592 in a local tissue engineering model, its potential systemic effects should also not be ignored. Thus, we performed HIF-1α IHC staining in rat kidney, which is a representative organ to study the systemic effect of HIF stabilizer^15^ (Fig. 10A). No significant differences in the number of HIF-1α (+) cells were observed among all three groups, which indicated that the locally administered FG-4592 in our model does not trigger systemic hypoxia responses (Fig. 10C).

## Discussion

Hypoxia-inducible factor is critical in the regulation of vascularization process in both physiological and pathological conditions^6^. Stabilization of HIF is an effective strategy to promote vascularization. Previous studies reported HIF stabilizers include CoCl_2_ and DFX, two representative ferrous ion antagonist, or DMOG, a competitive inhibitor of Prolyl hydroxylase. However, CoCl_2_ is highly toxic to humans and DFX compromises iron metabolism. DMOG is a promising drug which has been extensively studied^15,21,22^. But, DMOG may potentially inhibit other members of 2 oxoglutarate-dependent dioxygenases and cause off-target side effects^23^. FG-4592, a novel HIF stabilizer developed using structure-based drug design and high throughput screening technology, can selectively inhibit the HIF prolyl hydroxylases. FG-4592 was primarily designed to treat anemia since it can promote erythropoiesis through elevation of erythropoietin. The therapeutic effect of FG-4592 on anemia has been extensively studied, including through a FDA registered Phase III clinical trial for chronic renal anemia^19^. Here we reported for the first time that FG-4592 possess proangiogenic ability both *in vitro* and *in vivo* based on a subcutaneous chamber model. Our results might extend the utility of FG-4592 in tissue engineering and other fields in which extensive local vascularization is required such as wound healing and fat grafting. The systemic effects of orally administered FG-4592 like the generation of erythrocytes and side effects like transient abnormal liver function have been reported in the aforementioned clinical trials. The aim of this study was to explore the localized proangiogenic effect of FG-4592 in a tissue engineering setting. Therefore, we selected a subcutaneous chamber drug loading model to minimize the systemic influence on the body. From a safety point of view, we still studied the protein expression of HIF-1α in the rat kidney and found that it was not changed after FG-4592 subcutaneous administration, which confirmed that FG-4592 does not exert systemic effect in our model.

To verify whether FG-4592 could stabilize HIF-1α in our experimental settings, we detected the transcriptional and translational expression of HIF-1α and its downstream molecule VEGF, a growth factor which has been proven to have a strong proangiogenic effect^24^. Based on western immunoblot and Q-rt PCR analysis, the mRNA and protein level of HIF-1α and VEGF were upregulated, whereas HIF-1α was slightly decreased at the mRNA level. The upregulation of these molecules was in line with the HIF-1α protein stabilizing the effect of FG-4592. As far as we know, FG-4592 has no direct effects on the transcription of HIF-1α, thus the decrease in HIF-1α mRNA might be attributed to the negative feedback of elevated HIF-1α protein.

Fibrin gel has already been reported to deliver growth factors^25^ and reserve endothelial progenitor cells^26^ required during vascularization. Here we reported for the first time that fibrin gel could act as a small molecule drug delivery system to facilitate the local administration of FG-4592 and promote angiogenesis in subcutaneous chamber model. Since fibrin undergoes fibrinolysis and degradation over time, we used aprotinin to slow down fibrinolysis and prolong the lifespan of the fibrin scaffold. It would be interesting to study whether the combination of FG-4592 and angiogenic cells such as endothelial progenitor cells could synergistically enhance the vascularization process *in vivo*. It is worthy to note that the fundamental purpose of this study was to determine the proangiogenic effect of FG-4592, its optimal dosage and action time rather than directly creating a well-vascularized tissue engineering construct. Thus, we selected a well-established subcutaneous chamber fibrin gel drug delivery model, which created an isolated environment to eliminate other confounding factors and assess the effect of FG-4592. Nevertheless, some other scaffolds such as sponge materials or strategies such as arterial-venous loop (AV loop) may also generate a better-vascularized tissue engineering construct^27^.

We found that the thickness of the fibrovascular tissue was significantly increased in FG-4592 loading group in a dose-dependent manner, which indicated that a stronger neovascularization process was triggered after drug treatment. Fibrovascular tissue which consists of inflammatory cells, fibroblasts, and blood vessels is regarded as an indicator of the vascularization process in subcutaneous chamber tissue engineering model. A thicker layer of fibrovascular tissue indicates stronger neovascularization^3,26^. Since the hypoxic pathway has been implicated in inflammatory response^28^, we evaluated the inflammatory response by detecting pro-inflammatory cytokines expression in RAW264.7 cell line and by MAC2 macrophage staining *in vivo* after FG-4592 excitation. The transcriptional expression of TNFα in RAW264.7 remains unchanged after FG-4592 excitation whereas a slight increase of IL1β expression was observed. Since the increase of IL1β in a typical inflammatory response would be as high as thousands of times, our study indicated that FG-4592 does not exert pro-inflammatory effect *in vitro*^29^.The level of macrophage infiltration was similar among all groups, indicating that there was a baseline inflammatory response in our model which was not associated with the administrated drugs. Considering the exogenous origin of the tissue engineering chamber and fibrin gel, a certain level of inflammatory response was expected.

The morphology of neovasculature was studied using CD31 endothelial cell staining. A counterintuitive decline in total vessel number was observed after 48mM/L FG-4592 locally treatment whereas the average vessel area was elevated. In other words, the body tended to transform neovasculatures into larger size rather than form more branched neovasculatures, indicating that maturation of neovasculature was triggered. A complete angiogenic process includes the formation of nascent vasculatures and their maturation transformations such as remodeling, pruning and stabilizing with the help of mural cells containing pericytes, mesenchymal cells and smooth muscle cells, which results in the formation of few maturate vessels with large diameter^4^. Besides, during the maturation of neovasculatures, endothelial progenitor cells (EPCs) undergo transformation from proliferation towards differentiation, which is reflected by the decrease in Ki67 and increase of differentiation markers^30^. HIF-1 has been reported to be a better strategy of promoting neovasculature formation and maturation compared to administering any of the angiogenic growth factors separately^31^. The fibrovascular tissue treated with FG-4592 had more HIF-1α and VEGF positive cells, which was in accordance with *in vitro* experiment results, further demonstrating the regulatory effect of FG-4592 on PHD-HIF-VEGF axis. This also supported our hypothesis that locally administered FG-4592 could influence the hypoxic response system *in vivo* and act as a proangiogenic agent in a tissue engineering setting.

Interestingly, the shape of the vessel section was different among the three groups. In drug-treated group, vessel section tended to be long and narrow, which contrasted with the round shaped vessel section observed in the control group. Histological section has already been used to primitively analysis the vessel shape and orientation^32,33^. In order to quantitatively describe the vessel orientation from histological observation, we introduced a novel statistical magnitude, vessel shape index (VSI), which was calculated by dividing the width of the vessel section by its length. A small VSI value indicates that the vessel section has a spindle-like morphology. Since the fibrin gel-muscle tissue complex was sectioned in a verticle plane, a spindle-like vessel section indicated that the neovasculature was growing in an upward orientation to the drug-loaded fibrin gel (Supplementary Fig. 1). We also observed that VSI decreased in the drug-treated group in a dose-dependent manner. Thus it is reasonable to speculate that the loading of FG-4592 into the fibrin gel scaffold might preferentially induce the growth of neovasculature towards the drug-rich zone. A more detailed study using angiography technology like Micro-CT is required to confirm this concept.

## Conclusion

Here we verified the pro-angiogenic role of FG-4592 in tube formation model *in vitro* and subcutaneous tissue engineering chamber model *in vivo*. The stabilization of HIF-1α and the subsequent activation of its downstream molecule VEGF were found to be associated with FG-4592-induced angiogenesis. *In vivo* application of FG-4592 generated mature and aligned neovasculature. The fibrin gel loading system for the delivery of FG-4592 could be used in tissue engineering and other fields where intensive neovasculature is required, such as wound healing and fat graft.

## Supplementary information

Supplementary Video 1. The regulation of Hypoxia Inducible Factor-1α.

Supplementary Video 2. The production of fibrin gel.

Dataset 1
