# Supplementary material for: The pro-angiogenic role of hypoxia inducible factor stabilizer FG-4592 and its application in an in vivo tissue engineering chamber model
Source: Sci Rep. 2019 Apr 15;9:6035. doi: 10.1038/s41598-019-41924-5 (PMC6465281; doi:10.1038/s41598-019-41924-5)
Supplement: Supplementary file 3 — Dataset 1 [file 41598_2019_41924_MOESM3_ESM.pdf]

Title

The pro-angiogenic role of hypoxia inducible factor stabilizer FG-4592 and its application in an in vivo tissue engineering chamber model

Authors

Muran Zhou[1], Jinfei Hou[1], Yuan Li[1], Shan Mou[1], Zhenxing Wang[1], Raymund E Horch[2], Jiaming Sun[1], Quan Yuan[1]

Affiliations

[1]Department of Plastic Surgery, Union Hospital, Tongji Medical College, Huazhong University of Science and Technology,1277 JieFang Avenue, Wuhan 430022, China

[2]Department of Plastic and Hand Surgery, University Hospital of Erlangen, Friedrich Alexander University, Erlangen Nuernberg, (FAU), Germany

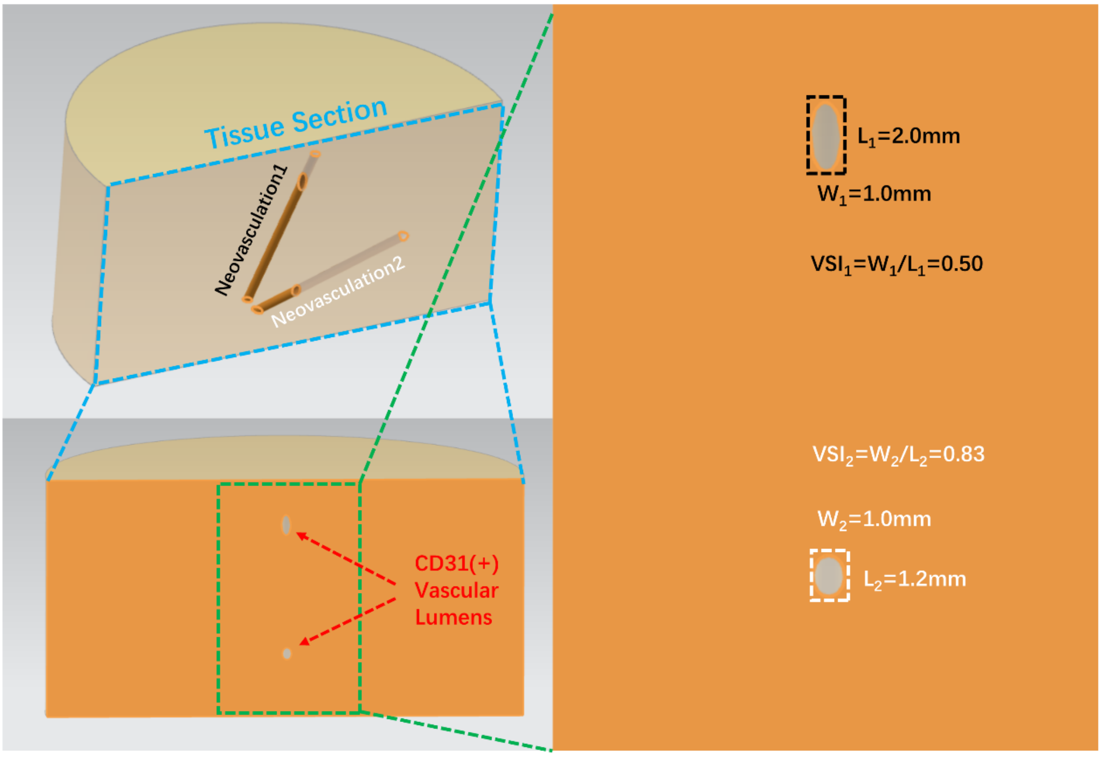

Supplementary Figure 1. The description of vessel shape index (VSI)

During the tissue sectioning procedures, neovasculatures in the fibrovascular tissue were cross-cut and the ellipse shaped vascular sections were colorized after CD31 immunohistological staining process. As a morphometric analysis method, we introduced vessel shape index (VSI), which was calculated by dividing the width (W) of the vessel section by its length (L), The smaller the VSI value, the slenderer the vascular section, and the VSI value provides information on whether the vascular tended to grow upwards or sideways.

Supplementary Video 1. The regulation of Hypoxia Inducible Factor-1α.

Supplementary Video 2. The production of fibrin gel.
